# Heterogeneous carbon/apatite composite adsorbent: cooperative ciprofloxacin adsorption

**DOI:** 10.1007/s11356-026-38338-y

**Published:** 2026-10-27

**Authors:** Schaline Winck Alberti, Fabiano Bisinella Scheufele, Gabriel Fiori Kruger, Leandro Couto da Silva, Edson Antônio da Silva

**Affiliations:** 1 Department of Chemical Engineering, Graduate Program in Chemical Engineering, Universidade Estadual Do Oeste Do Paraná (UNIOESTE), Toledo, Paraná Brazil; 2 Graduate Program of Chemical and Biotechnological Processes (PPGQB), Universidade Tecnológica Federal Do Paraná (UTFPR), Toledo, Paraná Brazil

**Keywords:** Emerging pollutants, Fluoroquinolone antibiotics, Environments, Wastewater treatment, Fish scale waste, Adsorption, Pore filling

## Abstract

This work aimed to evaluate the removal of ciprofloxacin (CIP) from aqueous solutions by adsorption onto a carbon/apatite composite (CApC) produced from fish scales. Initially, the adsorption of CIP was analyzed at different pH values, identifying pH 8 as the optimal condition (q_max_ = 79.09 mg g^−1^—experimental). Subsequently, kinetics and equilibrium studies were conducted in a batch system. The CApC material was characterized by scanning electron microscopy (SEM), Fourier-transform infrared spectroscopy (FTIR), X-ray diffraction (XRD), and separate adsorption tests of its carbonaceous and inorganic phases. The kinetic data were described using the pseudo-first-order model with two sites and diffusion in the pores, indicating limitation in the transport of the molecule. The Langmuir-Dubinin Radushkevich model (L-DR) provided the best fit to the data and predicted a total capacity of q_maxL-DR_ = 84 mg g^−1^, suggesting monolayer surface adsorption followed by micropore filling. Moreover, the adsorption mechanism is controlled by both surface-chemical interactions (i.e., hydrogen bonding and π-π stacking) and textural properties, wherein a tailored micropores size allows the “pore filling effect” retention mechanism for the CIP molecule. In general, the CApC adsorbent material showed efficiency and desirable characteristics for the treatment of water contaminated with the CIP drug.

**Supplementary information:**

The online version contains supplementary material available at https://doi.org/10.1007/s11356-026-38338-y.

## Introduction

Wastewater treatment is essential for water purification, environmental protection, and drinking water availability, particularly because of the increasing presence of emerging pollutants such as pharmaceutical waste, pesticides, heavy metals, and dyes associated with industrial growth. Materials such as activated carbon, zeolites, clays, and biomass composites are widely used due to their high adsorption capacity and effectiveness in removing micropollutants at very low concentrations (Finčur et al. 2021; Almeida-Naranjo et al. 2023; Kathi and El Din Mahmoud 2024) . Among emerging pollutants, antibiotics are of particular concern because their inadequate disposal and insufficient removal in wastewater treatment plants contributes to their spread in water bodies, favoring the development of antibiotic-resistant bacteria and causing ecosystem imbalances (Kulik et al. 2023; Samrot et al. 2023; Lima et al. 2024). In particular, ciprofloxacin residues may cause ecotoxicological effects on aquatic organisms and alter microbial communities. Moreover, its occurrence in wastewater and surface waters may contribute to the selection and dissemination of fluoroquinolone-resistant bacteria, increasing environmental and public health concerns (Dionísio et al. 2020; Diniz et al. 2021; Hayes et al. 2022).

Given this scenario, the search for efficient methods for the removal of drugs in aqueous media is essential and adsorption stands out due to its versatility, efficiency, and easy operation, especially in the highly diluted systems typical of emerging micropollutants (Ilavský and Barloková 2023; Bajda et al. 2024). In this context, scales from Nile tilapia (*Oreochromis niloticus*) emerge as an attractive alternative because they are an abundant and underutilized biomaterial. Fish scales comprise two main phases in their composition, an organic phase composed of collagen and an inorganic phase consisting of hydroxyapatite. Hydroxyapatite can adsorb both inorganic species and organic pollutants through ion substitution and surface interactions. Collagen is biocompatible, favoring the production of carbonaceous materials (Luo et al. 2020; Zhang and He 2023; Islam et al. 2024).

Sittitut et al. (2022) investigated a carbon/hydroxyapatite nanocomposite derived from fish scales, focusing on phosphorus (P) dissolution. The authors recognized the biphasic nature of the material, identifying hydroxyapatite-rich regions and predominantly carbonaceous regions. Although these regions were structurally examined, their individual contributions to contaminant adsorption were neither evaluated nor compared. Ji et al. (2020), in turn, prepared activated carbon from fish scales and identified phosphorus in the form of phosphates in the final material, which was attributed to the hydroxyapatite originally present in the raw material. However, the adsorbent was treated as a single material, without specific investigation of its distinct phases. Similarly, Latupeirissa et al. (2024), characterizing activated carbon produced from fish scales, reported the presence of two phases, described by the authors as graphite and carbon. This finding further indicates that fish scales can give rise to a structurally multiphase adsorbent; however, the adsorption capacity of each phase was not evaluated separately. Despite previous studies reporting the preparation of composite materials from fish scales, limited attention has been given to determining the individual contributions of the carbonaceous and apatite-derived phases.

Building on the previously reported preparation and textural characterization of the CApC material for 2,4-D adsorption (Alberti et al. 2023), the present study investigated its interaction with ciprofloxacin (CIP), a structurally larger, ionizable pharmaceutical contaminant whose adsorption is pH-dependent. The novelty of this work lies in the separate evaluation of investigating the heterogeneous carbon/apatite composite by separately evaluating its carbonaceous and inorganic fractions to quantify their respective contributions to CIP adsorption. Given this scenario, this work aims to evaluate the efficiency and elucidate the adsorption mechanisms of ciprofloxacin (CIP) onto a carbon/apatite composite (CApC). To achieve this, the synthesized material was characterized morphologically and structurally. The effects of pH, adsorption kinetics, and equilibrium were investigated experimentally and analyzed using mathematical models. Thus, we seek not only to demonstrate the adsorbent’s viability but also to understand the contribution of each of its constituent phases and the predominant mechanisms in the removal process of this contaminant.

## Materials and methods

### Materials

#### Obtaining and preparing carbon/apatite adsorbent

The Nile Tilapia (*Oreochromis niloticus*) scales were donated by COPISCES Cooperative, located in Toledo, Paraná, Brazil. The procedure of washing, storage, and preparation of the adsorbent composite material was carried out according to Alberti et al. (2023), called CApC. Briefly, the CApC was obtained by pre-carbonizing the scales at 330°C in an oxidative atmosphere, followed by chemical activation with KOH (1:1) at 950°C under a N_2_ atmosphere, washing with HCl (5 M) and water, and subsequent drying.

#### Adsorbate

The ciprofloxacin used in the experiments had a purity of 98.54% and was obtained from a pharmaceutical compounding company, located in the city of Guarapuava/PR. The concentration of the antibiotic in the liquid phase was determined by UV-Vis molecular absorption spectrophotometry (Genesys 10UV, Scanning); with the aid of quartz cuvette, scans were performed in the maximum molecular absorption range in the UV region with different concentrations of solutions (1, 3, 5, 8, and 10 mg L^−1^) and at different pH values (4, 7, 8, 9, and 10) in order to analyze the profile and the dependence of the pH and thus obtained the calibration curves for quantitation of CIP, within the linear range according to Lambert–Beer law, λ = 200 to 350 nm.

### Selection of the pH

Adsorption experiments were performed with two methods, with and without pH control to evaluate the dynamic process. The experiments were conducted at pH values of 4, 7, and 10 (pH control during the experiment was by adding acid solution HCl (1 mol L^−1^) or basic NaOH (1 mol L^−1^). Erlenmeyer flasks of 125 mL containing 50 mL of ciprofloxacin solution (50 mg L^−1^) with previously adjusted pH were placed in contact with 50 mg of activated carbon. All experiments were conducted in an orbital shaker at 30°C for 24 h to reach the equilibrium. Aliquots were removed from the solution to determine the final concentration. They were filtered with nylon filters (25 mm and 0.45 µm) for carbon retention, and the residual concentration of ciprofloxacin in the liquid phase was determined by UV-Vis spectrophotometry. The amount of adsorbed ciprofloxacin was calculated using Eq. 1:

1 $$q=\frac{V\left({C}_{0}-{C}_{e}\right)}{m}$$

In which *q *is the concentration of solute adsorbed by the adsorbent (mg g^−1^); *C*_0_ and *C*_e_ are the initial and final solute concentrations in solution (mg L^−1^), respectively; *V* is the volume of solution (L); *m* is the mass of the adsorbent used in each test (g).

### Properties of the ciprofloxacin molecule

The molecular geometry, speciation, and solubility of ciprofloxacin were calculated using Marvin 17.28 (ChemAxon Ltd 2026).

### Characterization of the composite adsorbent

#### Scanning electron microscopy (SEM)

Micrographs of the *in natura* fish scales and produced activated carbon were obtained using a scanning electron microscope (SEM). The samples were covered with a thin layer of gold by a metallizer and mounted on a sample holder using carbon adhesive tape.

#### X-ray diffraction (XRD)

Since the CApC has two main phases (carbonaceous and inorganic), XRD analysis was performed for both phases (separated manually) to observe the composition of each and compare them. The analysis was performed in a Bruker diffractometer (D2-PHASER) under the following conditions: continuous scanning mode, Cu-Kα radiation, nickel filter, and operated at 30 kV and 10 mA. The scan was performed at 2θ, with a speed of 0.02° s^−1^ per step of 1.0 s in the range of 5 to 80°.

#### Fourier Transform infrared spectroscopy (FTIR)

The determination of the functional groups present on the surface of the adsorbent composite was performed using Fourier-transform infrared spectroscopy in the infrared region of the different phases of CApC (inorganic phase and carbonaceous phase) and the adsorbent composite after adsorption. The samples were ground and mixed with potassium bromide (KBr) and then compressed until a thin film formed. The analysis was performed in an infrared spectrophotometer with Fourier transform (FTIR) (PerkinElmer–Frontier) operating in the range of 4000 to 400 cm^−1^, at a resolution of 4.0 cm^−1^.

#### Adsorption test of the different phases of the adsorbent composite

In order to determine which phase of the adsorbent composite has the greatest adsorption capacity (inorganic phase or carbonaceous phase), an adsorption experiment was carried out for 48 h under the following conditions: 30°C, CIP solution at 200 mg L^−1^, 250 rpm in Erlenmeyer flasks of 250 mL containing 100 mL of solution of ciprofloxacin, and 100 mg of adsorbent material (separated phases—white (inorganic) and black (carbonaceous)). The predominant separation of phases was visual and performed manually.

### Adsorption kinetics

Adsorption kinetics experiments were conducted in an Erlenmeyer flask placed in a thermostatic bath for temperature control. Two initial concentrations of CIP (100 and 200 mg L^−1^) were tested to obtain the equilibrium time. Experiments were conducted using 1 g of CApC and 1 L of CIP solution at 30°C, pH 8, and approximately 700 rpm. During the kinetic experiments, samples were periodically collected at predetermined times during the 24 h of experiment. Subsequently, the samples were submitted to UV-Vis analysis for determination of remaining concentrations in the liquid phase. The amount adsorbed was calculated through Eq. 1. To investigate the dynamic behavior of the process, kinetic models were fitted to experimental data, namely, the pseudo-first order (PFO) (Lagergren 1898) and pseudo-second order (PSO) (Ho and McKay 1999), pseudo-first order model with two sites (PFOTS) (adapted from Lagergren., 1898), and diffusion in pores (DP) (Ruthven 1984), listed in Table 1. In these equations, q and q_e_ represent the amount of solute adsorbed per unit mass (mg g⁻^1^), k_1_ is the PFO rate constant (min⁻^1^); k_2_ is the PSO rate constant (g mg⁻^1^ min⁻^1^); and D_ef_ is the effective particle diffusion coefficient (m^2^ s^−1^).

**Table 1:** Description of the kinetic models used to fit experimental data

Kinetic model	Equations	Application
PFO	$q={q}_{e}(1-\mathrm{exp}\left(-{k}_{1}t\right))$	It applies when the rate is controlled by mass transfer on the external surface of the adsorbent, ideal for the initial phase of adsorption
PSO	$q=\frac{{q}_{e}^{2}{k}_{2}t}{1+{q}_{e}{k}_{2}}$	Used to describe adsorption kinetics governed by complex interactions, such as chemisorption, fitting the entire process well from the beginning to equilibrium
PFOTS	$q={q}_{1}\left(1-\mathrm{exp}\left(-{k}_{1}t\right)\right)+{q}_{2}\left(1-\mathrm{exp}\left(-{k}_{2}t\right)\right)$	An empirical model based on PFO, used for systems with two distinct rate steps, describing adsorption on heterogeneous surfaces that have two types of adsorption sites
DP	$\frac{\partial q}{\partial t}=\frac{1}{{r}^{2}}\frac{\partial }{\partial r}\left({r}^{2}{D}_{ef}\frac{\partial q}{\partial r}\right)$	Applied when the adsorption rate is limited by the transport of the solute into the material's pores

### Adsorption equilibrium

The equilibrium data were obtained by batch experiments, wherein CIP solutions with different concentrations of CIP (15, 30, 55, 75, 90, 130, 150, 165, 180 and 200 mg L^−1^) were prepared. In each 250-mL Erlenmeyer flask, 100 mL of ciprofloxacin solution was contacted with 100 mg of CApC for a period of 48 h (to ensure that equilibrium was reached under all investigated conditions) at 250 rpm and 30°C, at 4 different pH values (7, 8, 9, and 10). Initial and final concentrations of the solution of ciprofloxacin were determined and the amount adsorbed at equilibrium was calculated using Eq. (1). To investigate equilibrium behavior, isotherm models were fitted to the experimental data, namely, the two-site Sips model (Adapted from the Sips 1950), the Langmuir-Dubinin Radushkevich model (Módenes et al. 2017), and the Sigmoidal Langmuir model (Limousin et al. 2007) as presented in Table 2. The isotherm models describe surface affinity, capacity, and heterogeneity. In the two-site Sips model, q_m1_ and q_m2_ are the maximum capacities of each site (mg g^−1^), K_1_ and K_2_ are the affinity constants (L mg^−1^), and n_1_ and n_2_ quantify heterogeneity. The Langmuir–Dubinin–Radushkevich model combines Langmuir’s monolayer capacity q_mL_ (mg g^−1^) and affinity K_L_ (L mg^−1^) with Dubinin–Radushkevich’s micropore filling capacity q_mD_ (mg g^−1^) and energy parameter β (mol^2^ J^−2^). In the Sigmoidal Langmuir isotherm, q_max_ defines the maximum capacity (mg g^−1^), K_L_ indicates affinity, and the parameter S controls the sigmoidal transition, reflecting cooperative effects. The relatively high CIP concentrations used in the kinetic and equilibrium experiments were selected to approach CApC saturation and ensure reliable model parameter estimation, rather than to reproduce the trace levels typically found in environmental waters.

**Table 2:** Isotherm models and equations

Isotherms	Equations	Application
Sips with two sites	${q}_{e}=\frac{{q}_{m1}{{(K}_{1}{C}_{e})}^{{n}_{1}}}{1+{{(K}_{1}{C}_{e})}^{{n}_{1}}}+\frac{{q}_{m2}{{(K}_{2}{C}_{e})}^{{n}_{2}}}{1+{{(K}_{2}{C}_{e})}^{{n}_{2}}}$	A model based on the Sips isotherm, it is applied to heterogeneous systems that do not follow ideal Langmuir behavior, describing adsorption onto two different types of sites
Langmuir-Dubinin Radushkevich	${q}_{e}=\frac{{q}_{mL}{K}_{L}{C}_{e}}{1+({K}_{L}{C}_{e})}+$ ${q}_{mD}\mathrm{e}\mathrm{x}\mathrm{p}(-\beta ({RTln(1+\frac{1}{{C}_{e}}))}^{2})$	Applied to complex systems that exhibit multiple adsorption mechanisms, combining Langmuir (monolayer adsorption) and Dubinin–Radushkevich (micropore filling). It is useful for systems where there are different types of adsorption sites or phases
Langmuir Sigmoidal	${q}_{e}=\frac{{q}_{max}{K}_{L}{C}_{e}}{1+{K}_{L}{C}_{e}+\frac{S}{{C}_{e}}}$	Used to describe systems where interactions between adsorbed molecules (adsorbate–adsorbate) occur or when there is low affinity between the solute and the adsorbent, especially at low concentrations

## Results and discussion

### Geometry and ciprofloxacin speciation

**Fig. 1:**
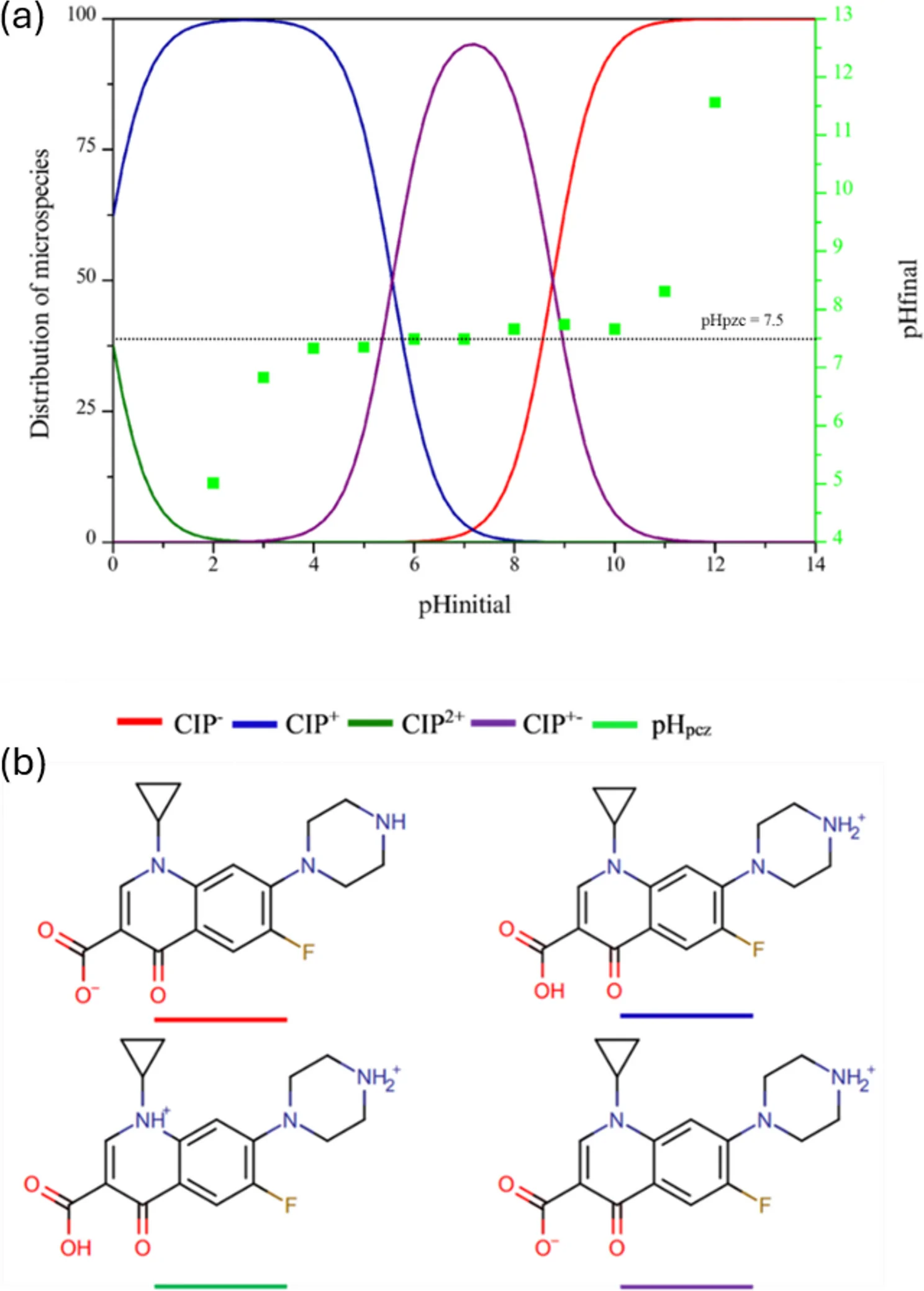
**a** pHpzc of the adsorbent composite and **b** dissociation of the ciprofloxacin molecule

The geometric parameters of ciprofloxacin and the speciation diagram as a function of pH were estimated (Fig. 1). The geometric parameters of the molecule were obtained: minimum and maximum projected molecular areas (43.14 and 96.6 Å^2^, respectively), as well as the molecular diameters: 5.02 Å (minimum) and 7.48 Å (maximum). The size of the molecule directly impacts the adsorption process, influencing the rate of adsorption when there is intraparticle transport and the geometry can impair the process if the pore diameter presents similar dimensions or even smaller (ChemAxon Ltd 2026).

Figure 1 presents the pH_pzc_ of CApC (pH_pzc_ = 7.5) together with the pH-dependent distribution of CIP microspecies. It is observed that there are different fractions of each species of CIP that vary according to the pH; four microspecies were considered: cationic, anionic, zwitterion, and neutral (pKa_1_ = 5.09 and pKa_2_ = 8.89) (Roca Jalil et al. 2015; Czyrski 2022). Based on the distribution of microspecies, it is possible to understand the affinity between the adsorbent and the adsorbate, explaining part of the adsorption mechanism. Therefore, three representative pH values were selected for investigation (4, 7, and 10).

### Analytical methodology

Based on the physicochemical properties and pH-dependent speciation of ciprofloxacin, an analytical methodology for CIP quantification by UV-Vis spectrometry was established.

The spectral scans revealed small shifts in the maximum absorption wavelength (see Figure S1 in the Supplementary Information), being necessary to construct different calibration curves: for pH 4 at the maximum absorption wavelength (*λ* = 278 nm) and Abs = 0.1209C–0.0286 and *R*^*2*^ = 0.99561; for pH 7 (at λ = 275 nm) and Abs = 0.095C–0.004 and *R*^*2*^ = 0.9997; for pH 8 (at λ = 271 nm) and Abs = 0.0969C + 0.01 and *R*^*2*^ = 0.99919; and for pH 9 and 10 (λ = 270 nm) and Abs = 0.0903C–0.006 and *R*^*2*^ = 0.99978. The calibration curves are provided in the Supplementary Information (see Figure S2).

This variation in the absorption peak occurs due to the distribution of microspecies, as seen in Fig. 1; UV-Vis absorption depends on the molecular electronic structure and ionization state. Thus, the absorption peak is influenced by the type of existing connection because changes in speciation alter the electronic transitions responsible for UV absorption. In addition, the absorption capacity at a given wavelength is altered if electrostatic interactions are occurring (Svanberg 2004; Langlois et al. 2005).

### Effect of the pH

The speciation of ciprofloxacin (CIP) is pH-dependent, as its molecule possesses two ionizable groups, allowing it to exist in cationic, anionic, or zwitterionic forms (Fig. 1). The adsorbent used in this study, CApC, was prepared via basic activation with KOH. This process introduces basic surface functionality groups onto its surface, as confirmed by FTIR analysis (see Sect. "Infrared spectroscopy with Fourier transform (FTIR)"). The interaction between the pH-dependent CIP species and the CApC surface suggests that adsorption is governed by a combination of electrostatic effects and non-electrostatic interactions, including hydrogen bonding, π-π interactions, and pore-related retention.

A significant challenge was the adsorbent’s tendency to alter the solution’s pH. In preliminary tests without pH adjustment, the initial pH increased from 4.0 to a final value of 7.89. In contrast, a control solution containing only CIP showed no significant pH change, as detailed in Table S1 (Supplementary Information). This effect is attributed to the release of ions, such as phosphate, from the composite material into the medium. To accurately assess the effect of pH on adsorption, a controlled experiment was conducted. Even with pH correction applied at intervals, the final pH only approximated the initial target value, underscoring the importance of continuous pH monitoring for precise analysis. It was noted that the adsorption capacity decreased when the experiment was conducted below pH 6, indicating this range is less favorable for CIP removal.

**Fig. 2:**
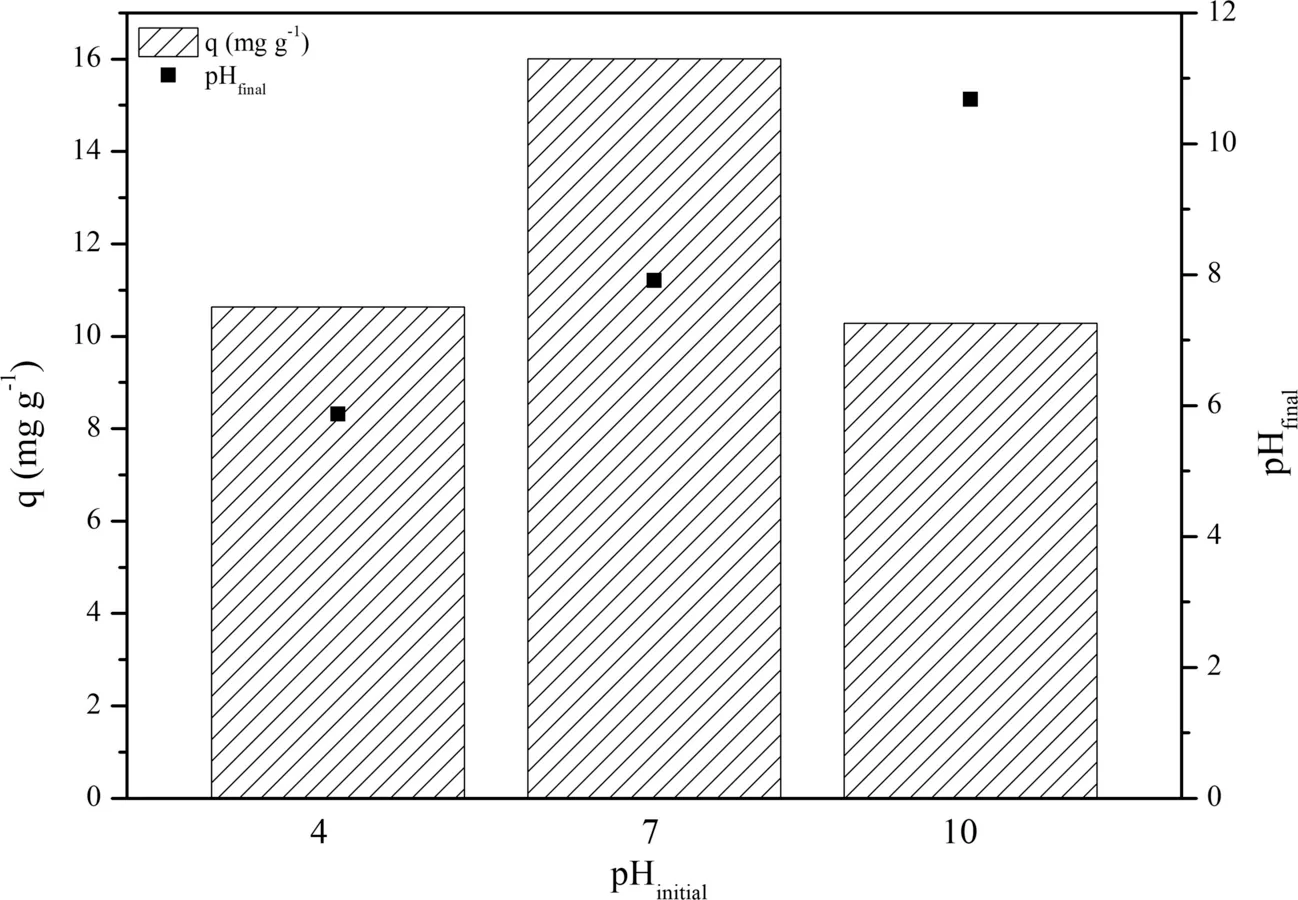
CIP adsorption capacity by the CApC material as a function of pH (Conditions: T = 30°C, ω = 250 rpm and t = 24 h)

Finally, to determine the optimal pH range, adsorption capacity was measured at three initial pH values: 4, 7, and 10. The results were 10.63 mg g^−1^ (at pH 4), 16.01 mg g^− 1^ (at pH 7), and 10.28 mg g^−1^ (at pH 10). As shown in Fig. 2, the highest adsorption capacity was achieved at an initial pH of 7, which resulted in a final pH near 8. Therefore, a pH range around 7.5–8.0 is considered ideal for the removal of ciprofloxacin with the CApC adsorbent. This optimal pH range was used in the subsequent adsorption experiments.

The point of zero charge (pH_pzc_) of the CApC adsorbent was determined to be 7.5. This indicates that the adsorbent’s surface is positively charged at pH < 7.5 and negatively charged at pH > 7.5. The speciation of ciprofloxacin is also pH-dependent: it is predominantly cationic (due to the NH_2_^+^ group) at acidic pH and anionic (due to the COO^−^ group) at basic pH. This explains the lower adsorption capacities observed at the extremes. At pH 4, electrostatic repulsion occurs between the positively charged adsorbent surface and the cationic CIP molecules. Similarly, at pH 10, repulsion occurs between the negatively charged surface and the anionic CIP molecules. The optimal adsorption occurred in a pH range around 8, close to the adsorbent’s pH_pzc_ (7.5). In this range, the adsorbent surface has a near-neutral charge, minimizing electrostatic repulsion. Furthermore, around this pH, ciprofloxacin coexists as multiple species (including the zwitterionic form), which can interact with the adsorbent surface through non-electrostatic mechanisms, leading to the highest removal efficiency.

This result is also practically significant, as pH values near neutrality are common in natural water bodies. The wide effective pH range demonstrates the operational versatility of CApC as an adsorbent for ciprofloxacin removal.

### Scanning electron microscopy (SEM)

**Fig. 3:**
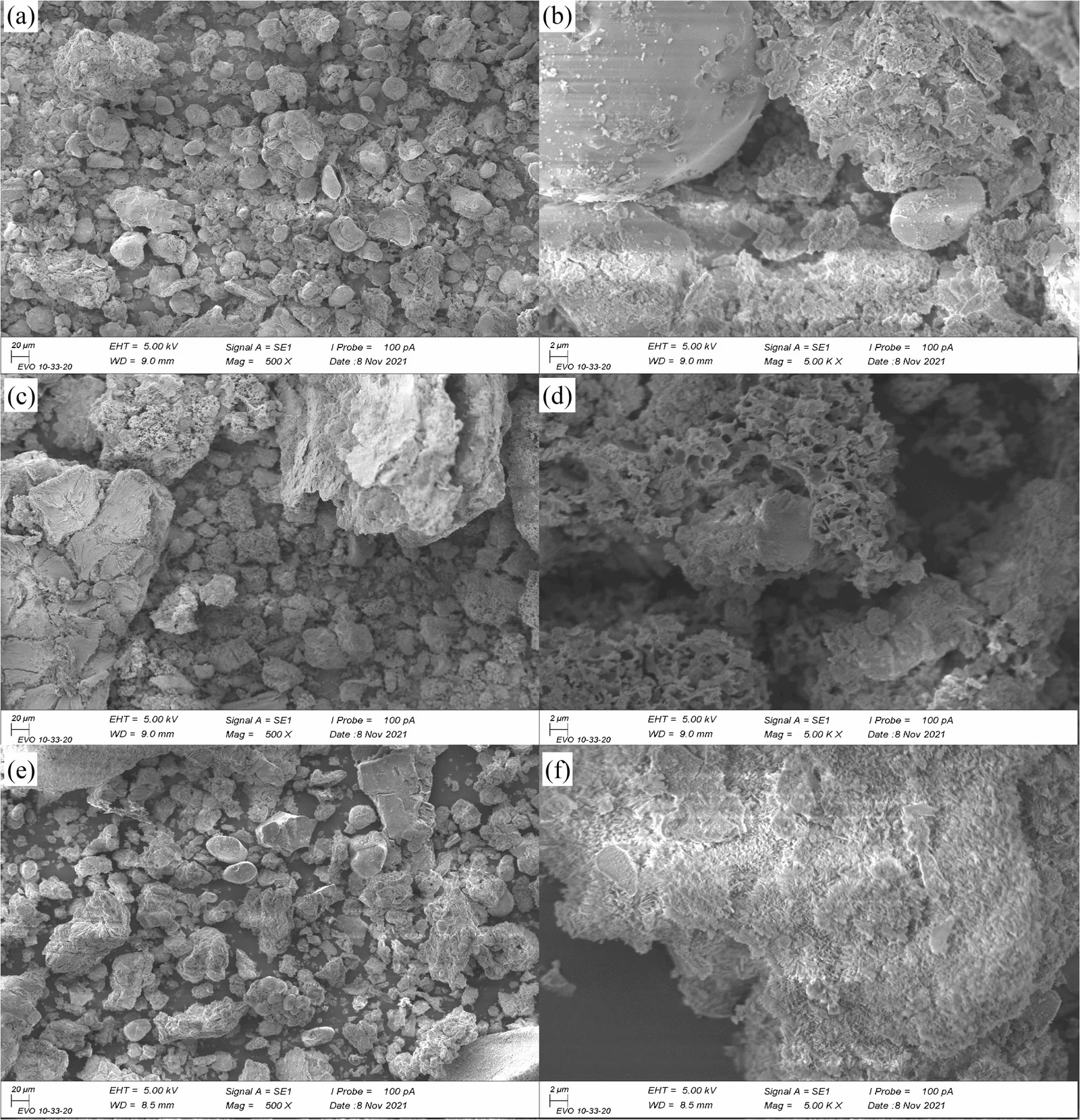
Electron microscopy of **a** inorganic phase in the × 500, **b** inorganic phase in the × 5.00 k, **c** carbonaceous phase in the × 500, **d** carbonaceous phase in the × 5.00 k, **e** CApC in the × 500, and **f** CApC in the × 5.00 k

Figure 3a–f shows SEM images of the inorganic phase (a, b), carbonaceous phase (c, d), and mixed CApC (e, f). The inorganic phase is denser, less porous, and rougher, whereas the carbonaceous phase is highly porous and irregular. Each fraction contains traces of the other, indicating incomplete separation. When the CApC is observed with both phases (mixed), the mixture is clearly perceived, resulting in an irregular, dense, and moderately porous carbon. This analysis is remarkable because the carbonaceous phase, derived largely from the collagen fraction of the scales, has a higher adsorption capacity, as evidenced in the section on the adsorption test of the different phases of the adsorbent composite. Furthermore, the results suggest that the inorganic phase of hydroxyapatite contributes more to the presence of functional groups than to the formation of a porous structure. This morphology is consistent with the textural properties of the CApC used in the present study, which were previously determined by N_2_ physisorption and reported by Alberti et al. (2023): BET-specific surface area of 72.13 m^2^ g^−1^, a total pore volume of 0.133 cm^3^ g^−1^, and a characteristic pore diameter of 22.36 Å (2.236 nm).

### X-ray diffraction (XRD)

**Fig. 4:**
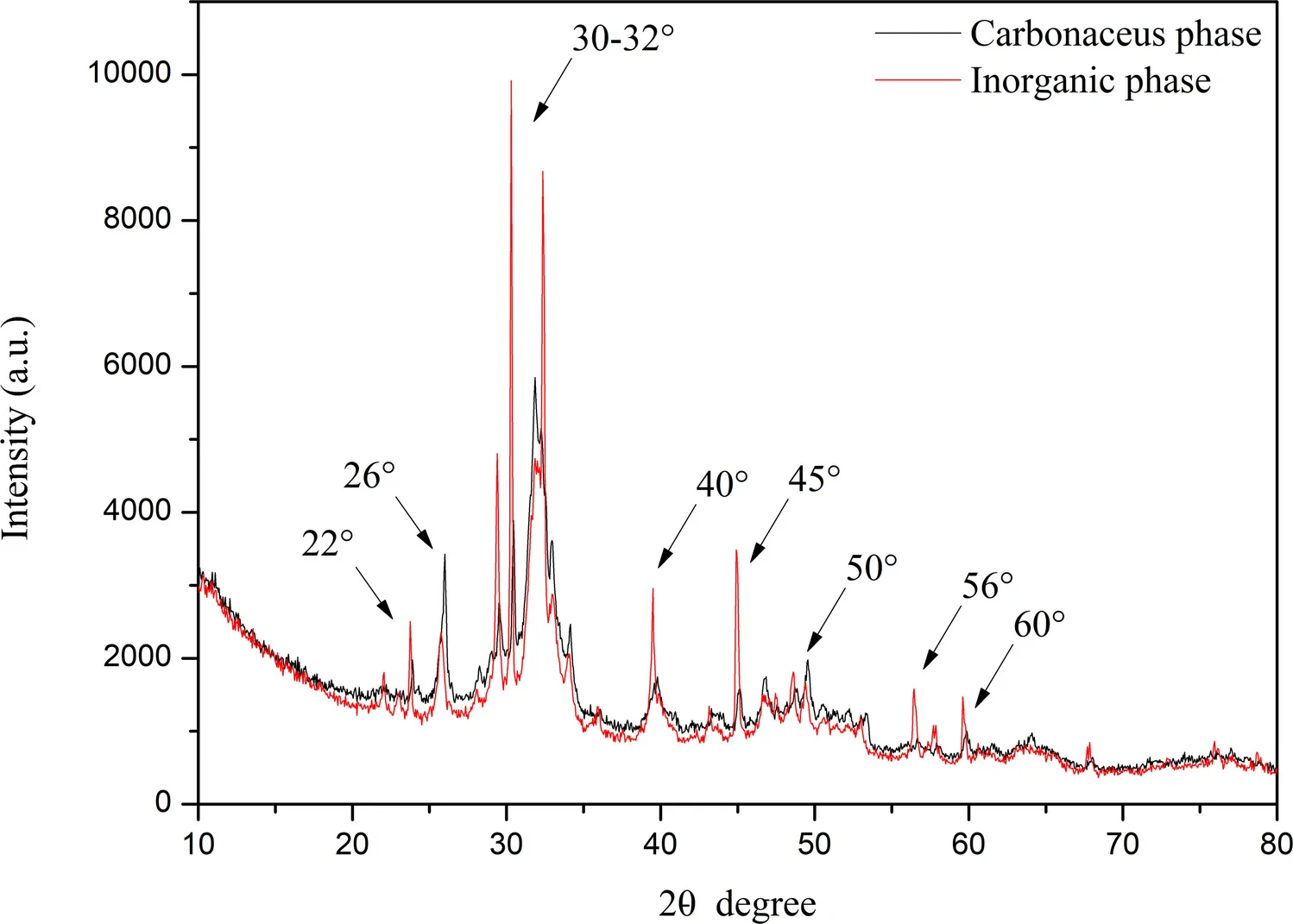
X-ray diffractogram of the carbonaceous and inorganic phases of the CApC sample

Figure 4 shows the XRD patterns of the carbonaceous and inorganic CApC.

The X-ray diffractograms for both the carbonaceous and inorganic phases exhibited several diffraction peaks. A broad feature centered around 22° is present in both patterns, which is a characteristic of the amorphous structure derived from the groups referring to hydroxyl and amino collagen present in the fish scales before carbonization, but now (after the carbonization) appears at a low intensity (Vieira et al. 2014). In the inorganic phase, sharp and intense peaks corresponding to the crystalline structure derived from hydroxyapatite (HAp) are clearly visible at 2θ = 30–32°, 40°, and 45°. The presence of these HAp peaks, potentially overlapping with signals from calcium carbonate as suggested by the FTIR analysis, confirms the inorganic nature of this phase. The other peaks at 26°, 56°, and 60° are associated with apatite domains exhibiting a low degree of crystallinity, consistent with the biogenic origin of the precursor material (Ikoma et al. 2003; Vieira et al. 2014). The coexistence of a broad amorphous halo (from collagen) and sharp crystalline peaks (from HAp) confirms that the CApC is an organic-inorganic composite material. Furthermore, the analysis shows that the phase separation was incomplete. The presence of the collagen peak in the inorganic phase pattern and residual apatite peaks in the carbonaceous phase pattern, indicates cross-contamination between the two separated materials.

### Infrared spectroscopy with Fourier transform (FTIR)

**Fig. 5:**
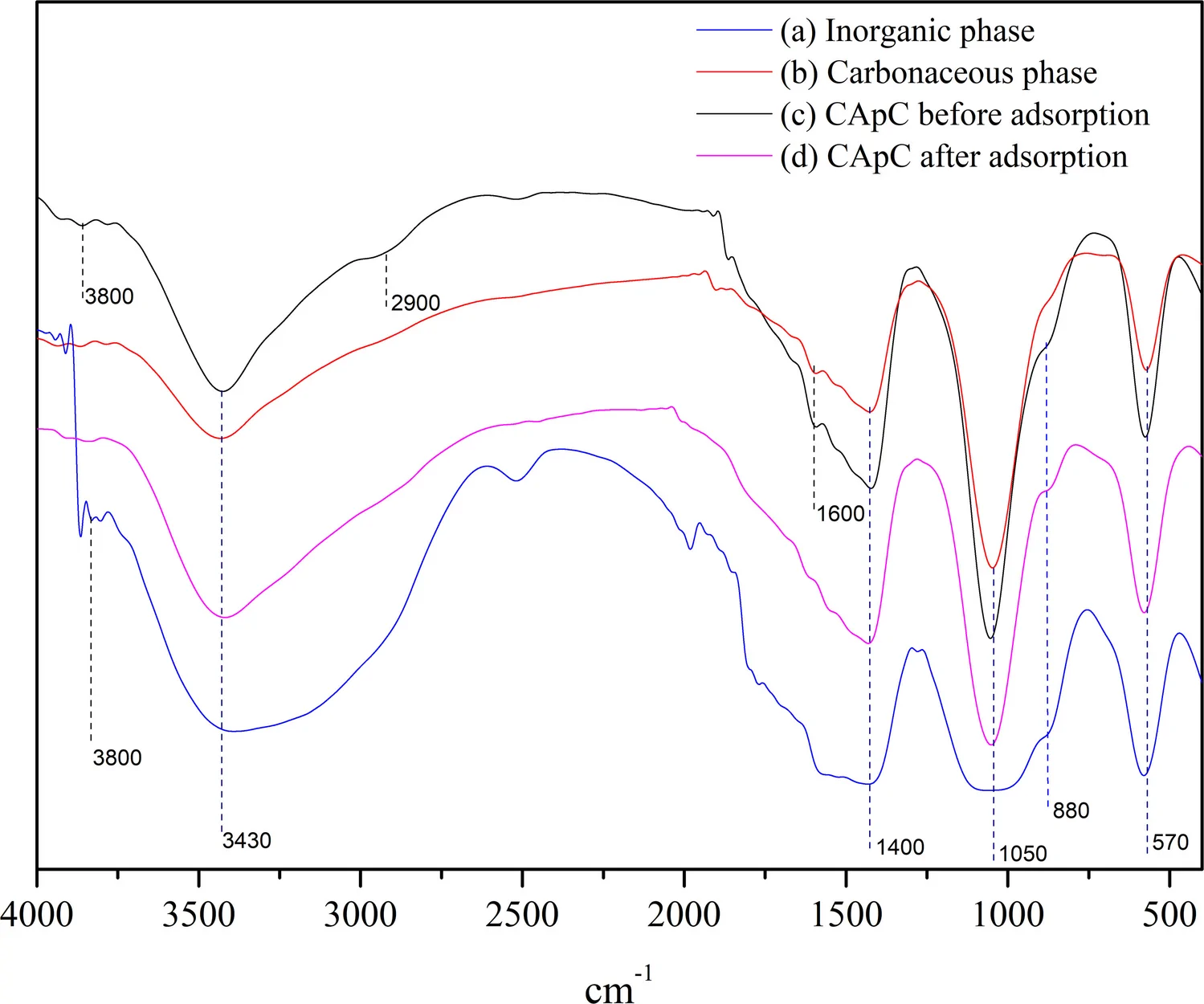
FTIR spectra of **a** inorganic phase, **b** carbonaceous phase, **c** CApC before adsorption, and **d** CApC after adsorption

Fourier-transform infrared (FTIR) spectroscopy was collected for the CApC composite adsorbent and its separate phases: (a) inorganic, (b) carbonaceous, (c) CApC before adsorption, and (d) CApC after adsorption, as shown in Fig. 5a, b, c, and d. The main objective was to chemically characterize the surface of the adsorbent and to investigate the possible molecular interactions involved in the ciprofloxacin (CIP) adsorption process. A summary of the identified functional groups is presented in Table 3.

**Table 3:** Functional groups identified by FTIR analysis

**Wavenumber (cm**^−1^)	**Groups**	**References**
3430–3800	O–H	Dovbeshko et al. (2000); Bin Mobarak et al. (2024)
2900	C-H	Zingare et al. (2022)
1600	C = C, C = O, carboxylic groups, amides	Smidt and Meissl (2007); Huang et al. (2014)
1400	Carbonate ions, carboxylic groups	Movasaghi et al. (2008); Huang et al. (2014)
1050	PO_4_^3−^	Ikoma et al. (2003); Bin Mobarak et al. (2024)
880	CO_3_^2−^	Bin Mobarak et al. (2024)
570	PO_4_^3−^	Ikoma et al. (2003); Zingare et al. (2022); Bin Mobarak et al. (2024)

Initially, a broad band centered at 3430 cm^−1^ is observed, which is a characteristic of the O-H stretching of hydroxyl groups from minerals and absorbed water. A weak band is also observed at 3800 cm^−1^ in the inorganic phase and less pronounced in spectrum (c), attributed to free O-H groups typical of hydroxyapatite (HAp). These hydroxyls can act as H-bond donors to the anionic carboxylate group (COO^−^) of CIP. In the 2900 cm^−1^ region of the CApC spectrum (c), the presence of aliphatic C-H groups is observed, attributed to the surface of the carbonaceous material present in the CApC.

The peaks at 1600 cm^−1^ and 1400 cm^−1^, present in the mixed and carbonaceous phases, can correspond to several overlapping groups related to aromatic rings, carboxylic acids, amides, and carbonates. The alteration in the bands within the 1600–1400 cm^−1^ region after adsorption (spectrum d) suggests that these groups participate in CIP adsorption. The peaks at 1050 cm^−1^ and 570 cm^−1^ are from phosphate stretching and bending modes, respectively, while the band at 880 cm^−1^ is due to carbonate vibrations. These three bands are signatures of HAp groups, confirming their structural presence in the composite. The elemental composition of fish scales is predominantly calcium and phosphorus, consistent with the hydroxyapatite mineral phase reported in previous studies (Ikoma et al. 2003; Bin Mobarak et al. 2024; Zingare et al. 2022). The FTIR phosphate bands (1050 and 570 cm^−1^) further confirm this composition, as detailed in Table 3.

Overall, the adsorption of ciprofloxacin onto the composite is driven by a combination of mechanisms, such as hydrogen bonding between the adsorbent’s hydroxyl groups and the drug’s carboxylate function, π-π stacking interactions involving the aromatic structures of both the carbon surface and the CIP molecule, and direct interactions at surface sites containing C-O, COO^−^, and nitrogen (Yasmin et al. 2024; Sharma et al. 2025).

### Adsorption test of the different composite phases

The synthesis yields a heterogeneous CApC material composed of two distinct phases, one carbonaceous and one inorganic, as well as regions where both coexist (see Figure S3 in Supplementary Information). To better understand the adsorption process, an adsorption test was performed with each of the phases to analyze which has the greatest adsorptive capacity. The fines were not removed due to the amount of carbon produced. The overall yield of CApC, considering all stages, is 38.27%. This yield was strongly affected by material loss during washing to constant pH.

**Fig. 6:**
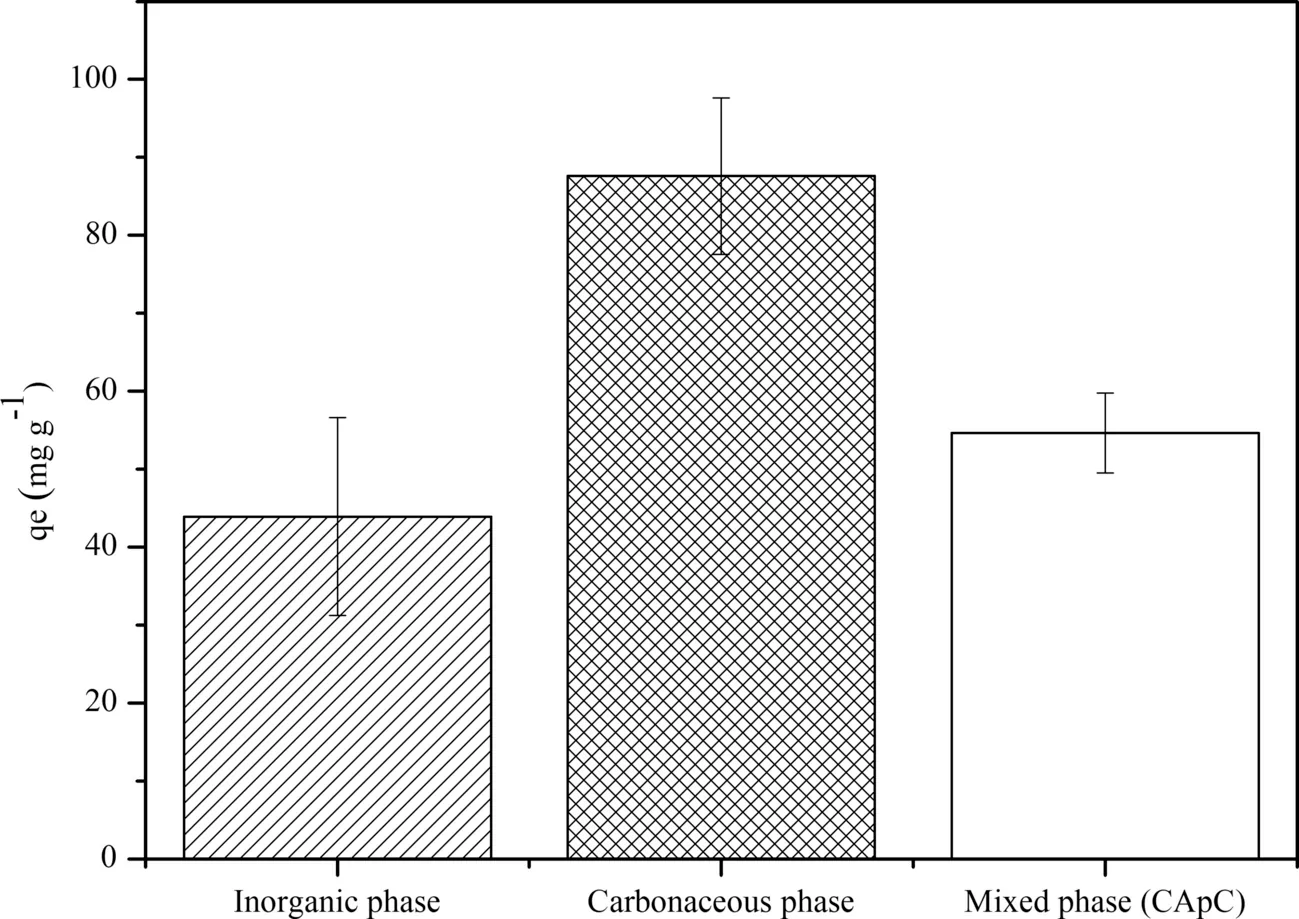
Adsorption capacity of the CApC phases (Conditions: T = 30°C, pH = 8, ω = 250 rpm, C = 200 mg L^−1^, and t = 48 h)

The composite presents two phases because the fish scales are composed of collagen and hydroxyapatite (HAp), resulting in the formation of two materials with distinct affinities for ciprofloxacin (CIP). HAp is known for its capacity to adsorb metal ions via ionic substitution within its crystal lattice (Ca^2+^, PO_4_^3−^, OH^−^). However, the primary adsorption mechanisms for an organic molecule like CIP involve surface interactions, such as hydrogen bonding and π-π stacking, which are limited on the predominantly ionic surface of the inorganic phase. In contrast, the carbonaceous fraction, with its aromatic nature, offered much more favorable adsorption sites, leading to a superior removal efficiency, as demonstrated in Fig. 6 (Anutrasakda et al. 2021; Zhang et al. 2021).

Collagen is widely used in the pharmaceutical and cosmetics industries and is a carbon-rich precursor with affinity for organic compounds (Huang et al. 2014; Indurkar et al. 2023; Dancila and Bosomoiu 2024).

The superiority of the carbonaceous phase is quantitatively evident: it adsorbed 87.6 mg g^−1^, whereas the inorganic phase removed only 43.9 mg g^−1^ (a capacity 50.1% lower). Although the exclusive use of the carbonaceous phase would be ideal for maximizing adsorption, its separation from hydroxyapatite is not a practical or viable process. Therefore, the CApC composite, which achieved a capacity of 54.65 mg g^−1^, represents the material’s realistic application. The FTIR results (Fig. 5) confirm that the CApC composite retains the essential functional groups from the carbonaceous phase (such as C-H and C = C), which are crucial for ciprofloxacin adsorption.

### Adsorption equilibrium

**Fig. 7:**
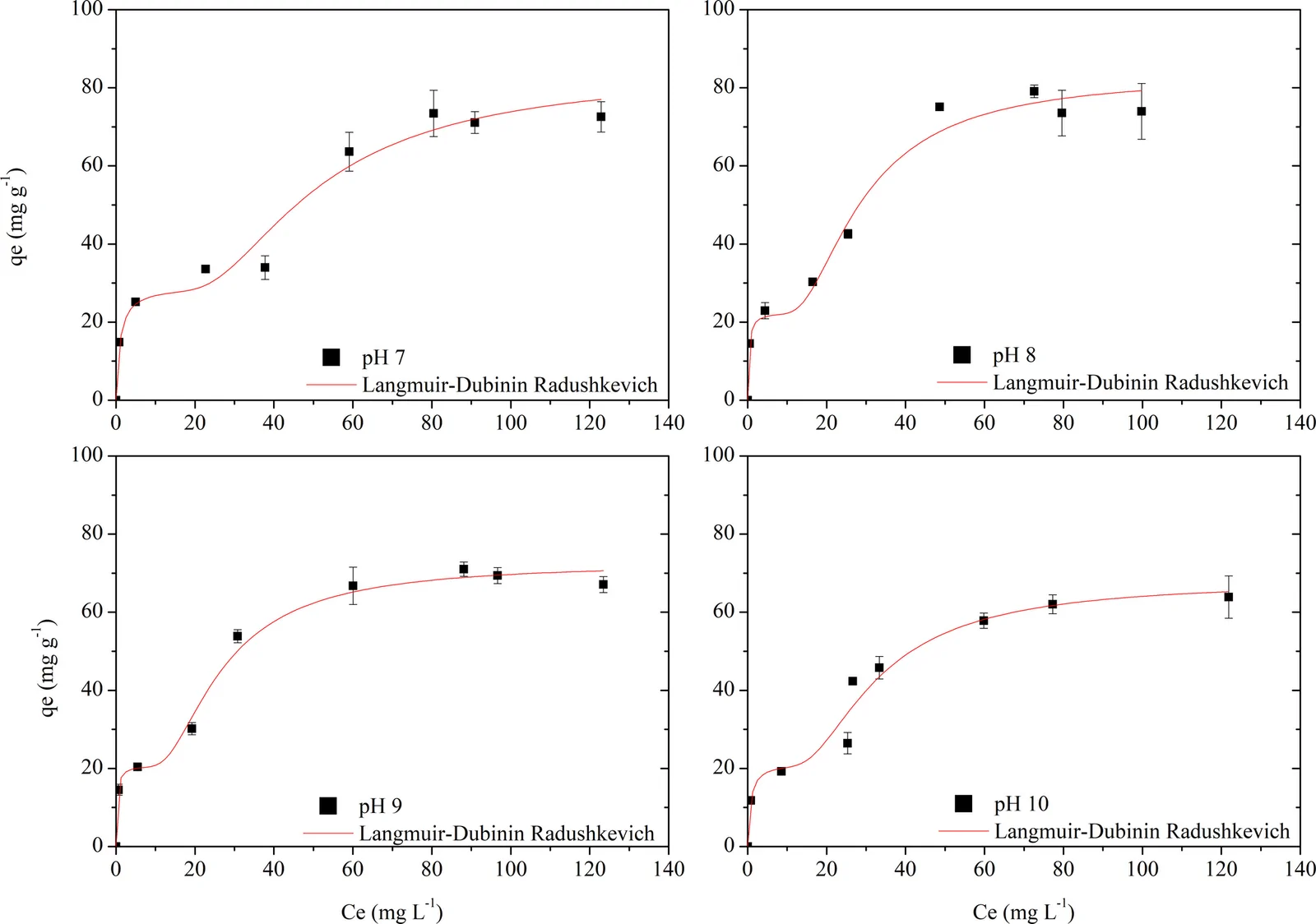
CIP adsorption equilibrium onto CApC material and isotherm models fitting at pH values 7, 8, 9 and 10 (Conditions: T = 30°C, ω = 250 rpm, and t = 48 h)

To better understand the adsorption mechanism, isotherm models were fitted to equilibrium experimental data, namely, Langmuir-Dubinin Radushkevich (L-DR), Sips with two sites, and Sigmoidal Langmuir, as presented in Table 4, together with the values of the estimated parameters. Figure 7 illustrates the experimental behavior of the equilibrium data under different pH conditions (7, 8, 9, and 10), together with fitted isotherm.

**Table 4:** Estimated parameters of the isotherm models fitted to the equilibrium data of the ciprofloxacin adsorption onto CApC material

Models	Parameters	pH 7	pH 8	pH 9	pH 10
**Langmuir-Dubinin Radushkevich (L-DR)**	**Q**_**mL**_** (mg g**^**−1**^**)**	**29.36**	**22.70**	**20.91**	**21.62**
	**K**_**L**_**(L mg**^**−1**^**)**	**1.04**	**3.56**	**4.27**	**1.47**
	**Q**_**mDR**_** (mg g**^**−1**^**)**	**54.65**	**60.39**	**51.55**	**46.28**
	**β (mol**^**2**^** J**^**−2**^**)**	**3.17 × 10**^**−4**^	**1.02 × 10**^**−4**^	**8.69 × 10**^**−5**^	**1.32 × 10**^**−4**^
	***R***^***2***^	**0.98**	**0.99**	**0.99**	**0.97**
	***χ***^***2***^	**5.77**	**4.24**	**1.70**	**4.40**
	**AIC**	**32.97**	**30.21**	**21.98**	**30.52**
**Sips with two sites**	q_m1_ (mg g^**−**1^)	16.41	19.56	19.92	9.90
	K_1_ (L mg^**−**1^)	2.35	5.29	8.54	4.03
	n_1_	3.56	4.78	3.74	0.84
	q_m2_ (mg g^**−**1^)	78.64	63.09	45.00	77.80
	K_2_ (L mg^**−**1^)	0.02	0.04	0.04	0.02
	n_2_	1.60	2.19	4.23	0.84
	*R*^*2*^	0.95	0.98	0.98	0.94
	*χ*^*2*^	12.11	6.62	4.76	10.43
	AIC	43.64	38.21	35.24	42.29
**Langmuir Sigmoidal**	q_max_ (mg g^**−**1^)	72.64	83.24	73.11	76.36
	K_L_ (L mg^**−**1^)	0.08	0.08	0.09	0.04
	S (mg L^**−**1^)	1.72 × 10^**−**5^	4.04 × 10^**−**4^	3.38 × 10^**−**4^	6.8 × 10^**−**4^
	*R*^*2*^	0.84	0.90	0.92	0.93
	*χ*^*2*^	38.59	30.42	18.34	11.92
	AIC	48.07	45.93	41.38	37.50

The values in bold indicate the isotherm model that best fitted the experimental data

Based on the coefficient of determination (R^2^), chi-square (χ^2^) values, and Akaike information criterion (AIC), the L-DR model showed the best fit across all analyzed conditions (*R*^*2*^ranging from 0.97 to 0.99). This model combines monolayer adsorption on homogeneous sites described by the Langmuir contribution, in which q_mL_ represents the limiting surface-adsorption capacity and K_L_ the apparent affinity constant, with micropore filling described by the Dubinin–Radushkevich contribution, in which q_mDR_ represents the limiting pore-filling capacity and *β* is related to the characteristic adsorption energy. This combination makes the model suitable for a heterogeneous and microporous material (S_micro_ = 90.28 m^2^ g^−1^) such as CApC. Thus, it adequately represents both the initial adsorption on high-affinity sites and the subsequent micropore-filling stage, which accounts for the sigmoidal isotherms observed (Fig. 7). Because the L-DR equation is additive, the asymptotic total capacity predicted by the model can be expressed as q_max,L-DR_ = q_mL_ + q_mDR_ and represents a model-derived total capacity.

The maximum adsorption capacity estimated by the L-DR model was obtained by summing the q_mL_ (monolayer) and q_mDR_ (micropore) parameters, resulting in total values (q_maxL-DR_) of 84.01 mg g^−1^ (pH 7), 83.09 mg g^−1^ (pH 8), 72.46 mg g^−1^ (pH 9), and 67.90 mg g^−1^ (pH 10). The maximum capacities obtained experimentally were 71.84 mg g^−1^ (pH 7), 79.09 mg g^−1^ (pH 8), 71.03 mg g^−1^ (pH 9), and 63.88 mg g^−1^ (pH 10). The adsorption capacities at pH 7 and 8 were the most prominent, indicating a higher interaction affinity. This is associated with the relationship between the solution pH and the pH_pzc_ (point of zero charge) of the activated carbon (CApC) at 7.5, which minimizes unfavorable electrostatic interactions. In this pH range, CIP occurs mainly in its zwitterionic form, favoring hydrogen bonding, π–π interactions, and pore-related retention.

Following the same rationale, as the pH increases, the CApC surface becomes negatively charged, while the anionic fraction of CIP increases, enhancing electrostatic repulsion (see Fig. 1). This change reduces the efficiency of occupation of the high-energy sites. Higher K_L_ values indicate a pH-dependent apparent affinity under experimental conditions. Finally, the low β parameter values (10^−4^ and 10^−5^) are consistent with the contribution of micropore-related adsorption, aligning with the characterization of the CApC (Saleh 2022; Agho and Archibong 2024).

Although the BET surface area of CApC (72.13 m^2^ g^−1^) is moderate compared with that of many conventional activated carbons, adsorption capacity is not controlled exclusively by the total surface area. The characteristic pore diameter of CApC is compatible with the molecular dimensions of CIP, favoring access to the internal pore structure and pore-related retention. In addition, the heterogeneous carbonaceous and apatite-derived phases provide chemically distinct adsorption sites capable of interacting with CIP through hydrogen bonding, π-π interactions, and other surface affinity mechanisms. The separate phase experiments confirmed that both fractions contributed to adsorption, although the carbonaceous fraction exhibited the highest capacity. Therefore, the relatively high CIP uptake can be attributed to the accessibility and affinity of the adsorption sites and to the cooperation between textural and surface-chemical properties, rather than to the BET surface area alone.

Comparing the Sigmoidal Langmuir and two-site Sips models, they exhibited lower *R*^*2*^values and higher AIC and *χ*^*2*^values, in addition to a weaker representation of the sigmoidal behavior. Thus, together with the FTIR, pH, textural, and separate phase adsorption results, the L-DR model supports a mixed adsorption mechanism involving surface adsorption followed by micropore filling.

Similar isotherm behavior has been reported for heterogeneous and multistep adsorption systems. Wernke et al. (2021) studied the adsorption of the antibiotic Cefalexin from aqueous media using activated carbon (composed of Ag and CuO nanoparticles decorated on graphene oxide) and obtained an isotherm with this behavior, which fit the Sips model (Wernke et al. 2021). Similarly, Soares et al. (2019) reported a sigmoidal isotherm for ciprofloxacin adsorption onto magnetic nanosorbents with siliceous hydro shells of alginic acid and carrageenan; the data were best described by the Dubinin–Radushkevich model. Likewise, Wang et al. (2020) obtained a sigmoidal isotherm for mercury removal using an iron sulfide composite supported on an Mg_2_Al-layered double hydroxide, which was adjusted by the Sigmoidal Langmuir model.

Regarding the adsorption capacities of ciprofloxacin previously reported in the literature, the following were found: Li et al. (2024) reported a CIP adsorption capacity of 384.4 mg g^−1^ at 25°C using activated carbon derived from waste cation-exchange resin; Essa et al. (2024) investigated CIP adsorption using a chitosan biocomposite intercalated in acid-activated calcium bentonite, reporting 50 mg g^−1^ at pH 5.5.

Among studies using fish scales as an adsorbent precursor, fluoride removal from groundwater using fish scale–derived biochar was reported, with an adsorption capacity of 6.3 mg g^−1^ at pH 4–6 (Kumari et al. 2026), utilizing activated carbon derived from fish scales to adsorb the dye methylene blue with a capacity of 119.04 mg g^−1^ (Yildiz and Yuksel 2023).

### Adsorption kinetics

**Fig. 8:**
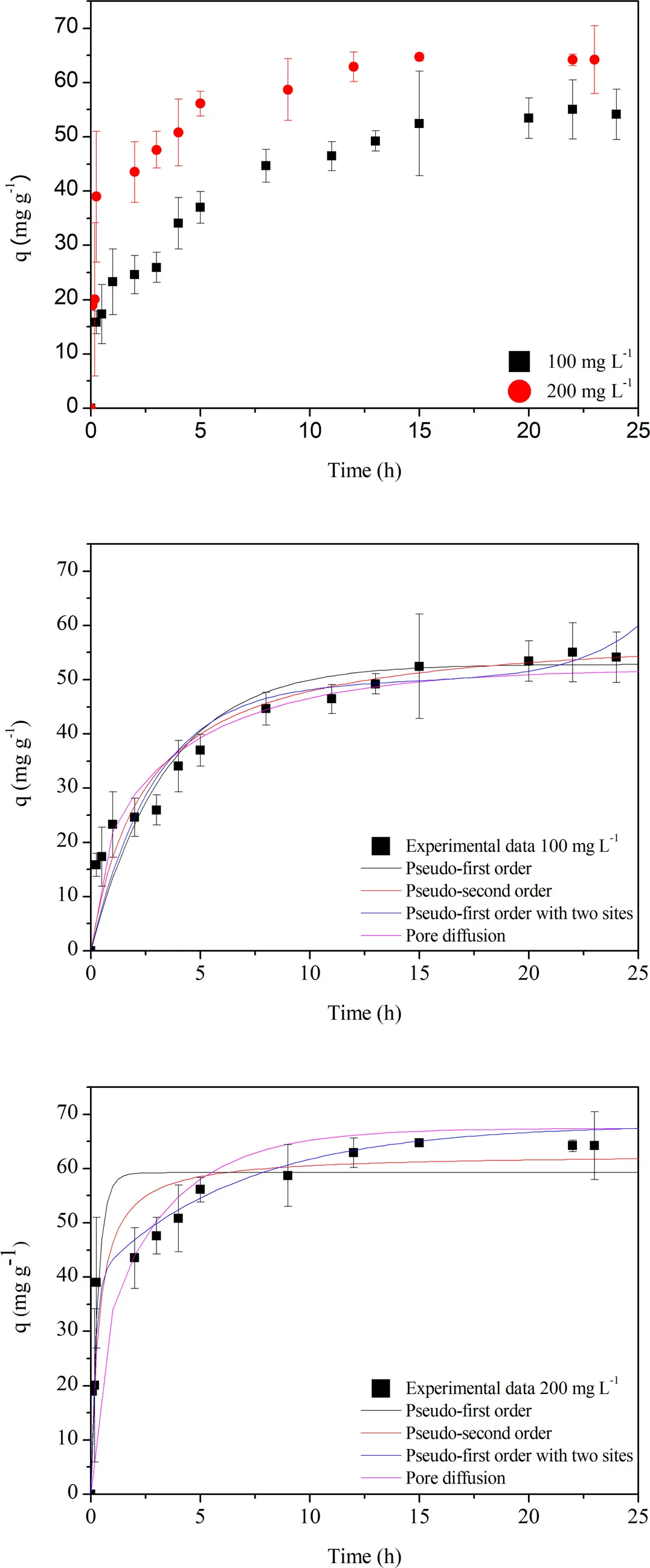
**a** Experimental kinetic data for ciprofloxacin adsorption onto CApC at 100 mg L^−1^ and 200 mg L^−1^; **b** kinetic modeling with the experimental data at 100 mg L^−1^; and **c** kinetic model fits to the experimental data at 200 mg L^−1^ (Conditions: T = 30°C, pH = 8, ω = 700 rpm, m = 1 g, V = 1 L and t = 24 h)

Figure 8 a displays the adsorption kinetics of ciprofloxacin onto the CApC composite at initial concentrations of 100 and 200 mg L^−1^. At 100 mg L^−1^, the system reached equilibrium in 15 h, achieving an adsorption capacity (q_e_) of 55.0 mg g^−1^. Increasing the initial concentration to 200 mg L^−1^ enhanced the mass transfer driving force, leading to a faster equilibrium time of 12 h and a higher capacity of 64.7 mg g^−1^. The larger error bars observed are likely attributable to the heterogeneity of the composite material.

The surface charge of the CApC (pH_pzc_) and the pH-dependent speciation of ciprofloxacin and the surface charge of CApC define the favorable pH range, since both for pH below 6.5 and pH above 10 adsorbent–adsorbate affinity decreases. However, the near-neutral optimum is operationally advantageous, since there are no requirements for pH adjustment. The four kinetic models tested (pseudo-first order (PFO), pseudo-second order (PSO), pseudo-first order with two sites (PFOTS), and diffusion in pores (DP)) are shown in Fig. 8b and c, and the fitted parameters are listed in Table 5.

**Table 5:** Kinetic models fit to the experimental data for CIP adsorption onto CapC

	**PFO**	**PSO**	**PFOTS**	**DP**
C_0_ = 100 mg L^−1^				
q (mg g^−1^)	53.40	60.31	q_s1_ = 15.62 q_s2_ = 42.63	52.27
Rate constant	0.0059 min^**−**1^	1.13 × 10^**−**4^ g mg^**−**1^ min^**−**1^	k_s1_ = 0.1726 min^**−**1^ k_s2_ = 0.0023 min^**−**1^	–
Diffusivity	–	–	–	1.75 × 10^**−**13^ m^2^ s^**−**1^
*R*^*2*^	0.9997	0.9998	0.9999	0.9999
AIC	95.61	88.95	58.43	63.64
C_0_ = 200 mg L^−1^				
q (mg g^−1^)	63.21	66.66	q_s1_ = 0.0129 q_s2_ = 56.17	68.81
Rate constant	0.0120 min^**−**1^	3.69 × 10^**−**4^ g mg^−1^ min^−1^	k_s1_ = 4.84 × 10^**−**3^ min^**−**1^ k_s2_ = 3.11 × 10^**−**2^ min^**−**1^	–
Diffusivity	–	–	–	3.24 × 10^**−**13^ m^2^ s^**−**1^
*R*^*2*^	0.9997	0.9998	0.9998	0.9999
AIC	88.32	75.37	85.62	72.20

The statistical criteria presented in Table 5 indicate that model performance depended on the initial CIP concentration. At 100 mg L^−1^, the PFOTS model provided the best fit, with the lowest AIC value (58.43) and a high *R*^*2*^value (0.9999), supporting the occurrence of two adsorption rate contributions on the heterogeneous CApC surface. At 200 mg L^−1^, the pore-diffusion model showed the lowest AIC value (72.20) and the highest *R*^*2*^value (0.9999), indicating a greater contribution of intraparticle transport under the higher adsorbate loading. Thus, the PFOTS and pore-diffusion results jointly support the heterogeneous nature of CApC and the influence of internal diffusion on the adsorption process.

The pseudo-first-order model with two sites (PFOTS) is consistent with the heterogeneous nature of CApC, as observed by SEM and adsorption tests performed using separate phases. The faster adsorption contribution may be associated with the highly porous and easily accessible carbonaceous phase. On the other hand, the slower contribution may be related to less accessible sites, including those associated with the inorganic phase of hydroxyapatite, where adsorption may also be influenced by intraparticle diffusion, as supported by the pore-diffusion model.

The pore-diffusion model yielded an effective diffusivity of 1.75 × 10^−13^ m^2^ s^−1^ for 100 mg L^−1^ and 3.24 × 10^–13^ m^2^ s^−1^ for 200 mg L^−1^, being consistent with the concentrations. Pore diffusion follows Fick’s law; that is, the adsorbate diffuses through the liquid-filled pores of the adsorbent and is subsequently adsorbed at internal sites. These results suggest that intraparticle diffusion contributes to the rate limitation through transport in the pore liquid along the internal surface; all these statements are supported by N_2_ physisorption and the heterogeneous morphology observed by SEM (Weber and Smith 1986; Balarak et al. 2021).

As determined by N_2_ physisorption analysis, the characteristic pore diameter of CApC (Φ_µp_), obtained using the Dubinin–Radushkevich (DR) method, is 22.36 Å (2.236 nm). This value is consistent with the pore size distribution previously reported for this material in Alberti et al. (2023) and is approximately three times the maximum ciprofloxacin molecular diameter of 7.48 Å (Φ_CIP_) (see Sect. "Geometry and ciprofloxacin speciation"). The estimated effective diffusivity (1.75 × 10^−13^ m^2^ s^−1^ and 3.24 × 10^−13^ m^2^ s^−1^, for 100 and 200 mg L^−1^, respectively) indicates that the predominant transport mechanism is configurational diffusion. That is, diffusion within the micropores constitutes the rate-limiting step, as the pore diameter is only marginally larger than the diameter of the adsorbed molecule. This interpretation is supported by the ratio between the micropore and CIP molecular diameters (Φ_µp_/Φ_CIP_), which is 2.99. A ratio between 1.7 and 3 (which can be extended up to 6 when regeneration is proposed) suggests that the adsorption process is influenced by pore size, due to the “pore filling effect.” Consequently, the intraparticle diffusion in micropores becomes the slow and controlling step of the entire adsorption process (Tang et al. 2018; Schneider et al. 2024).

The overall adsorption rate is governed by several factors, including concentration, temperature, and the adsorbent’s physical structure. At low concentrations, the process is primarily driven by the concentration gradient (the driving force) between the bulk solution and the adsorbent surface. Temperature influences adsorption kinetics by supplying thermal energy and reducing solution viscosity. Furthermore, the adsorbent’s porous structure provides significant mass transfer limitations to the system. The movement of adsorbate molecules into internal binding sites is subject to steric hindrance, causing diffusivity to be much lower, especially for large molecules navigating into narrow pores (Ruthven 1984; Pourhakkak et al. 2021). This is particularly relevant for the CApC composite, as the physisorption analysis revealed a significant micropore surface area of 90.28 m^2^ g^−1^. This finding confirms that intraparticle diffusion within these micropores is a key factor influencing the overall adsorption capacity.

Reusability is an important aspect for the practical application of adsorbent materials. Previous studies have reported varying degrees of adsorption performance retention over successive regeneration cycles for carbon-based and hydroxyapatite–carbon composite adsorbents. For instance, Ahmed et al. (2025) showed that the regeneration behavior of a hydroxyapatite–carbon nanocomposite depended strongly on the target contaminant, with nearly constant removal over four cycles for rhodamine B, whereas a decrease in performance was observed for levofloxacin. Similarly, recent studies using carbon-based adsorbents for CIP have demonstrated that adsorption performance can be partially retained after repeated adsorption–desorption cycles (Shen et al. 2026; saliq et al. 2026). These findings indicate that regeneration is feasible for related materials, but its efficiency depends on the adsorbent composition, adsorbate, and regeneration conditions. Therefore, dedicated regeneration experiments are required before conclusions regarding the long-term reusability of CApC for CIP removal can be established.

## Conclusion

The results indicate that CApC has potential for removing ciprofloxacin from aqueous media, demonstrating desirable characteristics for adsorbent material, such as favorable kinetics and adsorbate-adsorbent affinity. CApC achieved an experimental CIP adsorption capacity of 79.09 mg g^−1^, at pH 8.0 at 30°C, and remained effective over the pH range of 7–10. Its favorable performance under near-neutral conditions is practically relevant because it may reduce the need for extensive pH adjustment during water treatment. In addition, fish scales represent a promising precursor for the carbon/apatite composite adsorbent, contributing to the valorization of an abundant and underutilized residue from the fish-processing industry. Kinetics and equilibrium studies revealed the heterogeneous nature and the mixed mechanism of the adsorption process. The combined Langmuir-Dubinin Radushkevich (L-DR) isotherm successfully modeled the surface monolayer adsorption followed by micropore filling and provided a better statistical description than single-site or Sigmoidal Langmuir models. Meanwhile, the pseudo-first order with two sites (PFOTS) model clearly distinguished between a rapid adsorption phase (high-affinity and easily accessible sites) followed by a slower phase, limited by intraparticle diffusion within the micropores. The microporous structure is compatible with the molecular dimensions of CIP, allowing the “pore filling effect” retention mechanism that may contribute to the surface-chemistry affinity mechanisms (i.e., hydrogen bonding and π-π stacking). This reinforces the understanding of the coexistence of different mechanisms and interaction sites for complex adsorbent materials. However, the present study was conducted in batch systems using synthetic aqueous solutions and did not evaluate adsorbent regeneration, repeated adsorption cycles, competing species, real wastewater matrices, or continuous flow operation. In addition, the environmental and economic implications associated with KOH consumption, activation at 950°C, inert gas demand, acid washing, and water use still require further assessment. Future studies should therefore investigate CApC reuse, adsorption in complex aqueous matrices, fixed-bed operation, optimization of the activation conditions, and techno-economic and life cycle analyses before considering large-scale application.

## Supplementary information

Below is the link to the electronic supplementary material.

ESM 1 (PNG 86.7 KB)

(TIFF 22.7 MB)

ESM 2 (PNG 417 KB)

(TIFF 567 KB)

ESM 3 (PNG 1.50 MB)

(TIFF 418 KB)

ESM 4 (DOCX 1.27 MB)

## Data Availability

All data supporting the findings of this study are provided in the Supplementary Information. Additional datasets generated during the study are available from the corresponding author upon reasonable request.
