# Supplementary material for: Heterogeneous carbon/apatite composite adsorbent: cooperative ciprofloxacin adsorption
Source: Environ Sci Pollut Res Int. 2026 Oct 27;33(39):17798–815. doi: 10.1007/s11356-026-38338-y (PMC13740125; doi:10.1007/s11356-026-38338-y)
Supplement: Supplementary file 1 [file 11356_2026_38338_MOESM4_ESM.docx]

**Supplementary Information**

Additional data for use in this work

**Heterogeneous Carbon/Apatite Composite Adsorbent: Cooperative Ciprofloxacin Adsorption**

*Schaline Winck Alberti¹^*^, Fabiano Bisinella Scheufele^2^, Gabriel Fiori Kruger¹, Leandro Couto da Silva¹, Edson Antônio da Silva¹*

^*^Corresponding author: schaline.alberti@unioeste.br

Figure S1 Scanning of ciprofloxacin solutions at a concentration of 10 mg L^-1^ at different pH’s

**Figure S2** Ciprofloxacin calibration curves as a function of pH

Table *S1* Adsorption test with and without pH control. Conditions: pH 4, C=50 mg L^- 1^, T=30°C, V=50 ml, pH control with 30min, 2 h and 6 h of experiment, solution = no adsorbent composite material

| **pH Control** | **Test** | **m (g)** | **C (mg L^-1^)** | **Q (mg g^-1^)** | **pH_initial_** | **pH_final_** |
| --- | --- | --- | --- | --- | --- | --- |
| **No** | Solution | -- | 56.35 | -- | 4.0 | 5.52 |
|  | CApC | 0.0506 | 36.96 | 19.16 | 4.0 | 7.89 |
| **Yes** | Solution | -- | 46.09 | -- | 4.0 | 4.16 |
|  | CApC | 0.0546 | 34.38 | 10.72 | 4.0 | 5.87 |

Figure S3 Visual aspect of the CApC produced
